# Photonic Paint Developed with Metallic Three-Dimensional Photonic Crystals

**DOI:** 10.3390/ma5071196

**Published:** 2012-07-02

**Authors:** Po Sun, John D. Williams

**Affiliations:** Electrical and Computer Engineering Department, University of Alabama in Huntsville, Huntsville, AL 35899, USA; E-Mail: ps0004@uah.edu

**Keywords:** metallic photonic crystal, photonic paint, anti-counterfeit, tag and track, 3-D photonic crystal

## Abstract

This work details the design and simulation of an inconspicuous photonic paint that can be applied onto an object for anticounterfeit and tag, track, and locate (TTL) applications. The paint consists of three-dimensional metallic tilted woodpile photonic crystals embedded into a visible and infrared transparent polymer film, which can be applied to almost any surface. The tilted woodpile photonic crystals are designed with a specific pass band detectable at nearly all incident angles of light. When painted onto a surface, these crystals provide a unique reflective infra-red optical signature that can be easily observed and recorded to verify the location or contents of a package.

## 1. Introduction

Photonic crystals (PCs) have become a hot topic over the past decade as a new way to control the optical properties of materials [1,2]. These man-made microscopic periodic structures are optical analogues of the electronic band structure presented in semiconductors. If the dielectric constant is arrayed periodically in one dimension and homogeneous in the other two directions, it is a one-dimensional PC. Likewise, two- and three-dimensional PCs are structures whose dielectric constants are arranged periodically in two and three dimensions. Early work was performed on one- and two-dimensional PCs because the calculation and ease of fabrication allowed for a solid establishment of the field. As such, one-dimensional layered Bragg stacks have now matured into working devices [3,4,5,6], and many two-dimensional (2D) PCs are commonly applied in wave guides [7,8], fibers [9,10] and other photonic devices [11,12]. Unfortunately, the development of three-dimensional (3D) PCs is quite a bit more difficult. While some applications have been found for 3D PCs [13,14], these designs mainly focus on the application of defects in 3D PCs and the fabrication remains challenging and time consuming. For example, a 3D PC operating with greater than 85% efficiency requires three unit cells to be fabricated in each direction. For woodpile structures, this requires a minimum of 15 individual lithographic layers to be aligned, patterned, and either etched or plated [15]. However, recent improvements in interference lithography [16] and woodpile design [17] have allowed for the fabrication of three unit cell thick metallic PCs using single layer processes to be completed in days instead of weeks. To put 3D PCs into successful engineering applications, two conditions must be considered: (1) tunable optical properties interested in an application and (2) easy fabrication process.

This article presents a photonic paint design with tilted metallic woodpile PCs [18] and polyethylene, which can be fabricated easily and the interested optical properties of which are also easily tuned. The photonic paint utilizes 50–100 μm wide PCs mixed into a solution of polyethylene and toluene. The low viscosity polymer can then be applied to almost any surface by coating or spray techniques. As the solution dries, a relatively thin layer of polyethylene with randomly oriented 3D PCs remains on the surface. All that the observer sees is a clear plastic coating with small brown flecks of gold PC that reflect a very specific infrared (IR) signature. Each PC within the paint can be engineered such that the passband is constrained over a certain spectral region, thus providing a discrete signal that is not naturally occurring, difficult to fabricate, and not easily recognizable by unwanted eyes with night vision capability. Thus, we propose a new method for monitoring sensitive objects close-up or from a distance.

## 2. Results and Discussion

### 2.1. Woodpile Photonic Crystals in Free Space

**Figure 1:**
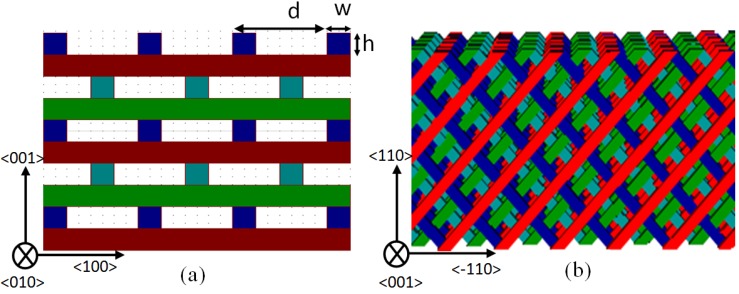
Side views of (**a**) the traditional woodpile photonic crystal structure and (**b**) the tilted-woodpile structure.

Figure 1(a) shows a traditional woodpile and its layer-by-layer periodic structure. Each layer consists of one-dimensional square logs with a distance (period in <100> and <010> direction) *d.* The logs have a square cross section with width *w* and height *h*. The traditional woodpile structure was fabricated with a layer-by-layer method [15] and the surface normal is along <001> direction. To simplify the fabrication, Toader and John proposed a tilted version of the woodpile [19]. Realization of this device in metals [17,18,20] has since led to an easily fabricated woodpile structure with some unique optical properties [21]. Woodpiles created by this method are essentially a rotated version of the same conventional woodpile, but the surface normal is along <110> direction. These tilted woodpiles are fabricated such that the top of the structure is oriented along <110> direction, allowing the device to be patterned in a single lithography and deposition process instead of the layer-by-layer sequence. As such, these devices can be generated in less time and for a significant reduction in cost. Figure 1(b) shows a side view of the tilted woodpile structure. With the top-down approach [18], a photonic crystal up to 2 square inches in size can be fabricated in hours or days rather than the weeks it takes to manufacture 15 layer metallic PCs.

For the photonic paint application, 50–100 μm wide PCs are achieved by patterning 50 μm wide grid lines into the X-ray mask in order to separate a single large area PC design into hundreds of small individual devices. The resulting tilted woodpile PCs can be lithographically patterned, plated, and released from the substrate using a sacrificial metal seed layer beneath the photoresist. The paint is generated by mixing the PCs uniformly into a solvated polymer.

**Figure 2:**
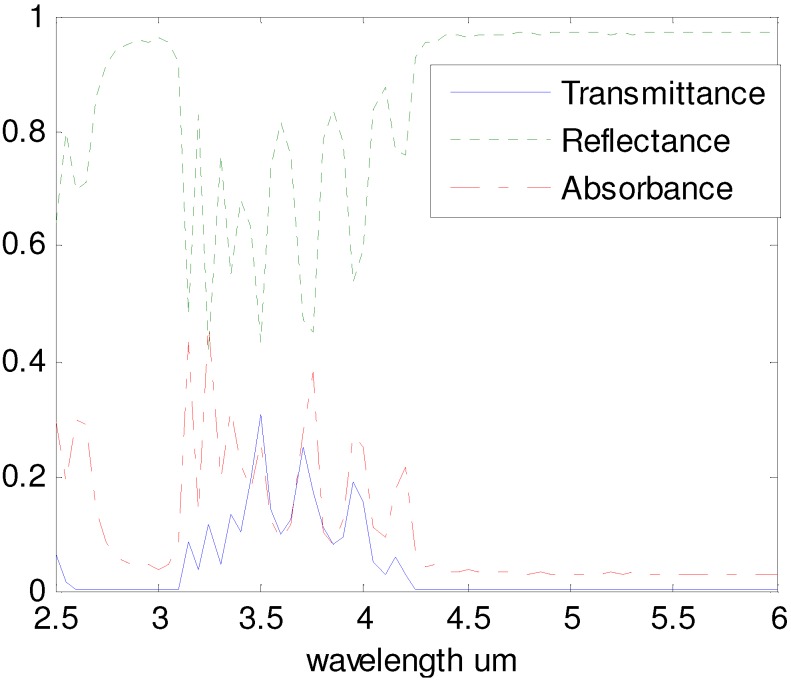
(Color online) Transmittance (blue solid line), reflectance (green dotted line), and absorbance (red dash-dot line) spectrum of a gold tilted-woodpile structure with *d* *=* 2 μm, *w* = 0.5 μm, *h* = 0.8 μm.

Further studies on the metallic tilted woodpile structure show that the forbidden bandgap can be interrupted by a passband beyond waveguide cutoff [21]. Figure 2 shows a numerical simulation representing the reflectance (green dotted line), absorbance (red dash-dot line), and transmittance (blue solid line) of a tilted-woodpile structure made of gold (the incident light is along <110> direction). In this article, gold is chosen as the material of woodpile photonic crystal because it can be readily electroplated, has excellent optical reflectivity, and demonstrates a high corrosion resistance. Other metals such as copper and nickel, which have similar spectral properties, can also be used.

One can see from the transmittance spectrum that there are two bandgaps, 2.6 μm to 3.1 μm and 4.3 μm to 6 μm, where no light can be transmitted. Between these two bandgaps is the passband, which is represented by a reflectance dip from 3.1 μm to 4.3 μm. The simulation presented in Figure 2 matches experimental results presented by Sun and Williams for fabricated tilted woodpiles of different beam dimensions [21]. The strong fluctuations appearing spectrally within the passband come from different plasmon polariton modes. In experimental measurements, these fluctuations are averaged out due to fabrication defects and the use of unpolarized light. The edge position and width of these passbands can be easily tailored for applications by changing the log size (*w* and *h*) of the woodpile structure. Using a zero alignment optical or X-ray lithography process, as demonstrated by Williams [18], slight changes in the 2D lithography mask design allow one to pattern discrete volumes of PC across several square millimeters, possibly centimeters, in size that have either different IR passbands or no passband at all.

The bandgaps and the passband of tilted woodpiles offer a new design parameter for 3D applications, allowing the creation of narrow and wide bandgaps whose position is designed to produce a specific dip in reflectivity at nearly any angle of incidence. Thus, the tilted woodpile can be designed to include spatially dependent reflectivity data when fabricated over large areas similar to that of a diffractive optic or, in the case of this article, provide a “fingerprint” by allowing the observer to probe the PC at both bandgaps and the dip simultaneously. The reflected light at these three wavelengths (two bandgaps and the reflectance dip) will provide a specific amplitude return that depends directly on submicron changes in the beam size of the individual unit cells.

### 2.2. Optical Properties of Tilted-Woodpile Structures in Polyethylene

**Figure 3:**
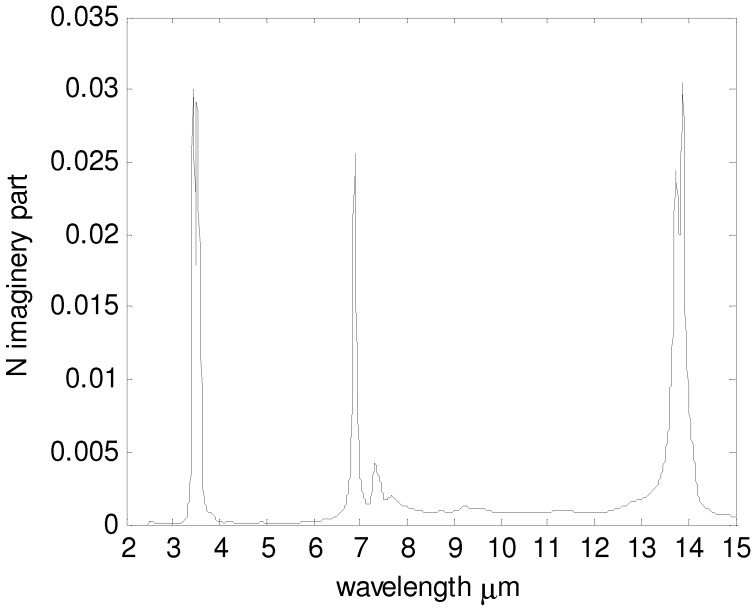
Imaginary part of the refractive index of polyethylene.

As important as the PC is to this design, the polymer matrix in which the PC will be suspended is equally so. It should be noted that the polymer will become the low index dielectric surrounding each gold beam that comprises the PC. The ideal background material should be transparent across the entire usable transmission spectrum, so that all absorption would come from PCs and not the background material. However, such polymers do not exist and one must choose from a selection of polymers with very little infrared fingerprint. For this effort, polyethylene was chosen over other materials for its transparency, biocompatibility, and costs. Other materials such as benzocyclobutene (BCB) might also be used, but cost 20 times more to produce and have not been as well characterized in the infra-red. Polyethylene is a commercial plastic with well-documented visible and IR transparency [22,23,24,25] with the exception of three distinct IR absorption peaks. These peaks must be accounted for when modeling the absorption of light within the PC and the remaining polymer film. It is commonly used for elastomeric diffractive optics, due to their chemical stability, insolubility to water, plasticity, and insensitivity at temperatures between −20 and 80 °C [26,27]. These properties make polyethylene good casting molds for our photonic signature. The real part of the refractive index of polyethylene in an infrared range between 1 and 10 μm changes very slightly and can be estimated as a constant 1.53 [22]. The absorption of polyethylene can be represented by the imaginary part of the refractive index [23,24], which is plotted in Figure 3. For most wavelengths, the absorption is small enough to ignore, except the three absorption peaks located at 3.5 μm, 6.9 μm and 13.9 μm. For this effort, the imaginary refractive index was tabulated and entered directly into the calculation of the photonic bandgap and all other absorption data presented in this article.

**Figure 4:**
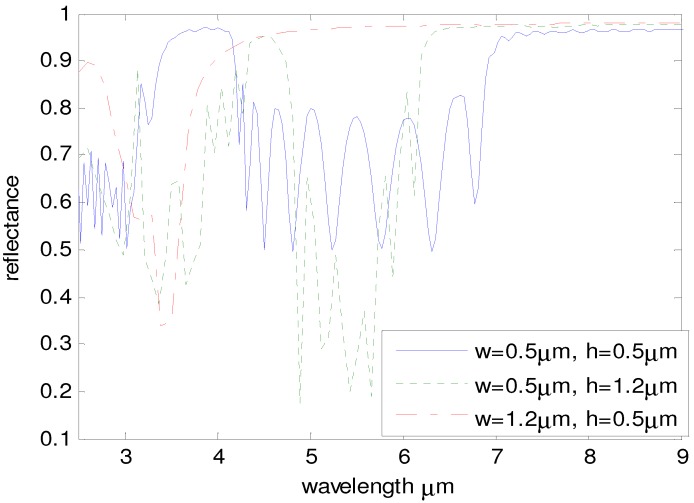
(Color online) The reflected spectrum of tilted woodpile PCs with different log size in a polyethylene dielectric: (1) blue solid line with *w* = 0.5 μm, *h* = 0.5 μm and* d* = 2 μm; (2) green dotted line with *w* = 0.5 μm, *h* = 1.2 μm and* d* = 2 μm; (3) red dash-dot line with *w* = 1.2 μm, *h* = 0.5 μm and* d* = 2 μm.

Designing woodpile PCs with different individual log sizes can give different reflected spectra, *i.e*., different tag information. For this study, the optical properties of tilted woodpile structures with different log sizes in polyethylene were simulated using R-Soft’s finite difference time domain package. Figure 4 shows the simulated reflected spectrum of tilted woodpile PCs made of gold logs with slightly different widths and heights. For this simulation, the dielectric surrounding the metal is polyethylene with the same thickness as the metallic pattern solved for (i.e. there is no polyethylene over or under the structure). We show that changing only the log width (*w*) and height (*h*) of woodpile structures can create very different optical passbands between 4 μm and 7 μm. For the red (dash-dot) reflectance line, no passband exists and the reflectance is about 1 μm from 4 μm to 8 μm; while for the blue (solid) reflectance line, the reflectance oscillates between 4 μm and 7 μm. Notice that for the red (dash-dot) reflectance line, the absorption peak of polyethylene at 6.9 μm does not affect the reflectance because the wavelength is located in the forbidden band and the EM wave is reflected without going through the paint film. While for the blue (solid) reflectance line, the absorption peak at 6.9 μm is located in the passband, so the EM wave passes through the paint film and is partially absorbed by polyethylene.

By changing the log width (*w*), height (*h*), and the distance between logs (*d-w*), the passband between 4 μm and 7 μm in Figure 4 can be designed to generate a unique optical signature specific to the application. The trend can be approximately described by this way: increasing the ratio *h/w* makes the passband deeper and narrower; increasing the distance between logs (*d-w*) will shift the right edge of the passband right, which makes the passband wider. When the distance (*d-w*) is small enough, the right edge of the passband will move to the left and meet the left edge, which means the passband disappears, just like the red (dash-dot) reflectance line.

### 2.3. Design of the Photonic Paint

**Figure 5:**
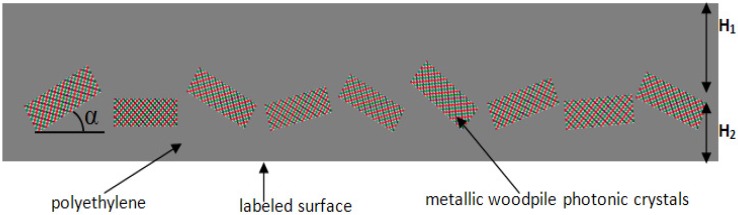
Schematic of the designed photonic paint where the metallic woodpile PCs are embedded into the polyethylene film. H_1_ and H_2 _are average distances from the PCs to the upper and bottom side of the polyethylene film; α is the tilted angle between the photonic crystal and the labeled surface.

The photonic paint consists of gold tilted woodpile PCs randomly mixed into the polyethylene film attached to any labeled surface, as shown in Figure 5. It is assumed that any coated surface will be painted such that individual PCs are randomly oriented within a thick polymer film of height H which consists of two partial heights H_1_ and H_2_. Here, H_2_ is designated as the median height from the base of the coating to the center of the average placement of PCs. Height H_1_ is the remaining thickness of the polymer coating.

**Figure 6:**
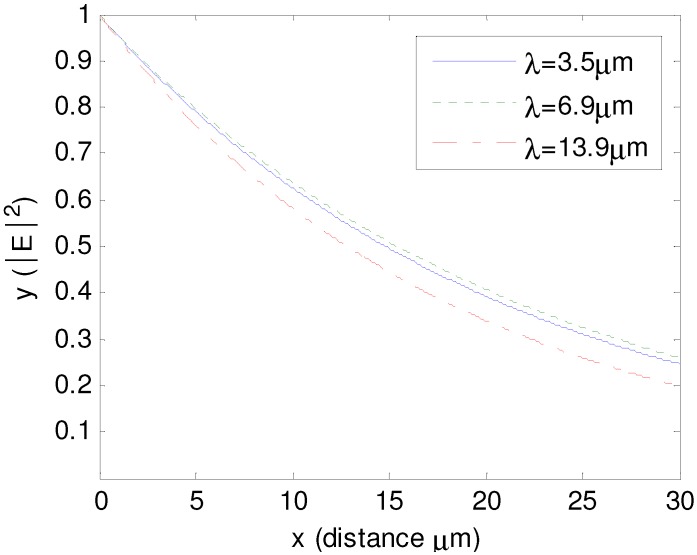
Decay curve of EM wave in polyethylene at the three absorption peaks 3.5 μm, 6.9 μm and 13.9 μm.

In the practical process of attaching the photonic paint to a surface, the thickness of either H_1_ or H_2_ cannot be zero so the effect of H_1_ and H_2 _must be considered. Further study, however, shows that the thickness below the tilted-woodpile PCs does not affect the reflected spectrum (or very little). This is because most EM waves are reflected by the PCs and cannot go into the area below the PCs. Therefore, only the effect of H_1_ needs to be considered here. For three absorption peaks of polyethylene, the imaginary refractive index is near 0.03. The decay curve of EM power is plotted in Figure 6, where the x-axis represents the propagation length in polyethylene and the y-axis represents EM power normalized to 1. This curve shows that when H_1_ = 6 μm (total travel distance is 12 μm), 50% of EM power is absorbed by the upper polyethylene layer at the three wavelengths. Thereby, if H_1 _is larger than 6 μm, there are always dips in the reflected spectrum at 3.5 μm, 6.9 μm and 13.9 μm. Thus, none of the three signal detection windows should be centered at these three wavelengths. Fortunately, the design is flexible enough to provide signal detection markers centered at different wavelengths.

**Figure 7:**
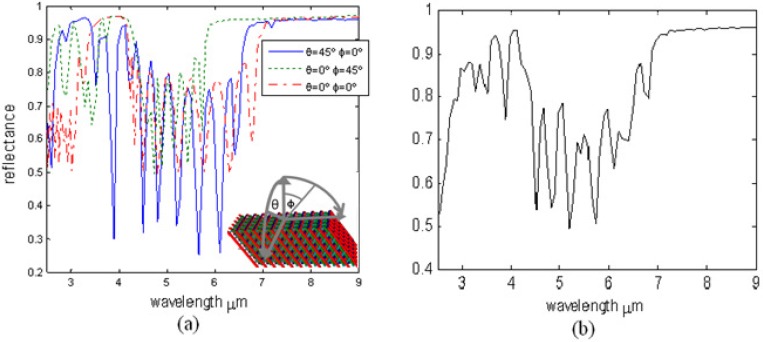
(Color online) (**a**) Three reflectance spectra of a gold tilted woodpile PC in polyethylene with different incident angles: (1) θ = 45° Φ = 0°, (2) θ = 0° Φ = 45 and (3) θ = 0° Φ = 0°; (**b**) the average reflectance of these three cases.

Since woodpile PCs are laid with random tilted angles (not necessary to be parallel to the surface), the effect of this random angle must be considered. Fortunately, optical properties of the metallic tilted-woodpile PC remain similar over a large solid angle, which means that even if light is launched in a large incident angle, the reflectance spectrum can still remain the specific character needed for the TTL application. In fact, light with very large incident angles are not necessarily included in this case. Notice that the effective reflecting surface area of the woodpile is *A × cos α*, where *A* is the normal surface (surface normal is along <110> direction) area and *α* is the tilted angle, as shown in Figure 5, which means increasing the tilted angle will decrease the effective reflecting area. Thereby, most collected light would come from PCs with a small tilted angle and it is sufficient to consider a 45° incident angle when designing the device. Figure 7(a) shows the reflectance spectra of a gold tilted woodpile PC in polyethylene with different incident light angles. Here, H_1_ is assumed to be zero; Φ and θ are angles in different incident planes (as shown in the subplot). All three cases have the reflective signature we want, *i.e*., two bandgaps and the reflectance dip (passband) between them. Although the right edge of the passband shifts with the change of the incident angle and the width of the passband also changes, the total effective reflectance of these three cases shows a very clear signature feature, shown in Figure 7(b). For these two figures, H_1_ is assumed to be zero. Although only three angle positions are selected here, these positions can represent significant angular changes of small PCs distributed within the paint. Furthermore, these calculations match previously published trends [18,21] regarding the simulated and measured response of tilted woodpile PCs at various angles of incidence that detail bandgap performance between normal incidence and a 60° angular offset. Figure 7(b) predicts an average response of the photonic paint. In reality, the reflectance dip should be smoother and have less fluctuation due to the random distribution of PCs. It is key to note that the photonic paint will have a reflectance peak followed by a partial transmission window, then a large reflective band which can be used as a three-wavelength optical signature. To achieve a unique optical signature, it is not necessary to use different PCs in the paint. Simply choose a desired optical signature that can be demonstrated using a single PC design which can then be fabricated and mixed into the polymer matrix. Only PCs with the same structural parameters randomly dispersed into the polyethylene matrix will give the desired unique spectral feature. However, the design response can be varied significantly by changing the scale size or the individual log size of the PC with a minimum achievable resolution of 0.1 μm using either Deep X-ray or interference beam lithography and electroplating.

Polarization of incident light often affects the reflectance of the photonic systems. For this article, all reflectance spectra presented are averaged results of both TE and TM modes. In fact, all simulations show that no matter which polarization the incident light is, the reflectance gives a similar bandgap-passband-bandgap character, so there is no special polarization requirement for the testing IR light source. Another factor affecting the reflectance spectrum in this design is the size and volume fraction of PCs in the polymer matrix. We suggest starting with 100 μm wide crystals that are three unit cells thick. The volume fraction used will depend on the application. Commercial tracking and anticounterfeit applications may want to cover more than 30% of the active area to improve signal to noise ratio. However, tracking objects indiscriminately will require significantly less surface coverage and a well-focused laser source and detector system.

## 3. Conclusions

This article has presented the design of a photonic paint with tilted metallic PCs and polyethylene, based on the unique bandgap-passband-bandgap character of tilted metallic woodpile PCs for anticounterfeit and TTL applications. Factors that may affect the reflective IR optical signature of the photonic paint have been discussed, including log size, absorption of polyethylene, light incident angles, polarization, and PC size. With this design, photonic paints with tunable and clear reflective signatures can be produced by a simple process.
